# Prediction of acute kidney injury in intensive care unit patients

**DOI:** 10.1186/s13054-018-2248-x

**Published:** 2018-11-16

**Authors:** Rui-Juan Guo, Fu-Shan Xue, Liu-Jia-Zi Shao

**Affiliations:** Department of Anesthesiology, Beijing Friendship Hospital, Capital Medical University, Beijing, 100050 People’s Republic of China

In their recent article assessing the predictive ability of urinary liver-type fatty-acid binding protein and serum N-terminal pro-B-type natriuretic peptide for acute kidney injury (AKI) in patients treated at a medical cardiac intensive care unit (ICU), Naruse et al. [1] did not provide any severity score, such as the APACHE II score or the SOFA score. The available evidence shows that patients’ severity of illness and level of organ failure upon admission to the ICU are independently associated with the occurrence of AKI [2, 3].

Furthermore, it was unclear whether the serum creatinine levels used for diagnosis of AKI had been corrected based on fluid balance. It has been shown that not adjusting serum creatinine levels for fluid balance can underestimate the incidence and severity of AKI in the ICU patients, as a positive fluid balance can dilute serum creatinine [4].

Finally, the discriminative ability of risk prediction models for AKI was assessed by c-statistic, but the calibration was not performed with the Hosmer-Lemeshow test. The calibration assesses the ability of a prediction model to match the number of actual events across deciles of risk-stratified subgroups. A *P* < 0.05 indicates poor calibration of the prediction model or a lack of fit between two models [5].

## Abbreviation

AKI: Acute kidney injury
